# RETRACTED: Li et al. Rheumatoid Arthritis-Associated MicroRNA-155 Targets SOCS1 and Upregulates TNF-α and IL-1β in PBMCs. *Int. J. Mol. Sci.* 2013, *14*, 23910–23921

**DOI:** 10.3390/ijms27083549

**Published:** 2026-04-16

**Authors:** Xiaochuan Li, Feng Tian, Fei Wang

**Affiliations:** The Department of Hand & Foot Surgery, Shengjing Hospital of China Medical University, 36 Sanhao Street, Heping District, Shenyang 110001, China; ftshjyy@163.com (F.T.); fwshjyy@163.com (F.W.)

The journal retracts the article titled, “Rheumatoid Arthritis-Associated MicroRNA-155 Targets SOCS1 and Upregulates TNF-α and IL-1β in PBMCs” [1], cited above.

Following publication, concerns were brought to the attention of the Editorial Office regarding the integrity of the images, the accuracy of the authorship list, and the origins of the study reported in this article [1].

In accordance with the journal’s procedures, the Editorial Office and Editorial Board conducted an investigation which identified indications of duplication between Figure 4E in [1] and Figure 5E in [2], despite these figures being presented as representing different experimental conditions. As these two articles were under submission at the same time by different authorship groups, this raised further concerns regarding the authenticity and provenance of the data, as well as the accuracy of the authorship. As a result, the Editorial Board has lost confidence in the reliability of the findings reported in this article and has decided to retract the publication.

This retraction was approved by the Editor-in-Chief of the *IJMS*.

The authors have been informed of this retraction but did not provide a comment on this decision.

## References

[1] Li X., Tian F., Wang F. (2013). RETRACTED: Rheumatoid Arthritis-Associated MicroRNA-155 Targets SOCS1 and Upregulates TNF-α and IL-1β in PBMCs. Int. J. Mol. Sci..

[2] Zhang Y., Chen N., Zhang J., Tong Y. (2013). Hsa-Let-7g miRNA Targets Caspase-3 and Inhibits the Apoptosis Induced by ox-LDL in Endothelial Cells. Int. J. Mol. Sci..

